# Child Salivary SIgA and Its Relationship to Enteric Infections and EED Biomarkers in Maputo, Mozambique

**DOI:** 10.3390/ijerph17093035

**Published:** 2020-04-27

**Authors:** Frederick G. B. Goddard, Jacqueline Knee, Trent Sumner, Rassul Nalá, Thomas Clasen, Joe Brown

**Affiliations:** 1 Gangarosa Department of Environmental Health, Rollins School of Public Health, Emory University, Atlanta, GA 30322, USA; frederick.goddard@emory.edu (F.G.B.G.); thomas.f.clasen@emory.edu (T.C.); 2 School of Civil and Environmental Engineering, Georgia Institute of Technology, Atlanta, GA 30332, USA; Jacqueline.Knee@lshtm.ac.uk (J.K.); trent.sumner@gmail.com (T.S.); 3 Department of Disease Control, London School of Hygiene & Tropical Medicine, London WC1E 7HT, UK; 4 Instituto Nacional de Saúde-Ministério da Saúde Maputo-Mozambique, C.P. 264, Mozambique; rassulmn@gmail.com

**Keywords:** salivary antibodies, immunology, infectious diseases, enteric pathogens, global health

## Abstract

Characterizing child immunological responses to enteric infections with antibody detection in serum can be challenging in resource-constrained field settings, because sample collection requires trained individuals and its invasive procedure may lead to low response rates, especially among children. Saliva may present a promising non-invasive alternative. The objectives of this research were to compare salivary antibody levels in children to enteric infections and biomarkers of environmental enteric dysfunction (EED). We collected saliva samples from children aged one to six years enrolled in a sanitation trial in Maputo, Mozambique, and characterized salivary secretory immunoglobulin A (SIgA) concentrations with enzyme-linked immunosorbent assays. We used multilevel linear models to analyze cross-sectional associations between salivary SIgA and the number of concurrent enteric pathogen infections, as well as EED biomarkers in matched stool samples. Median salivary SIgA concentrations in this study population were 54 μg/mL (inter-quartile range (IQR): 34, 85 μg/mL), and SIgA levels were similar between children of different ages. SIgA was lower in children experiencing a higher number of concurrent infections −0.04 log μg/mL (95% confidence interval (CI): −0.08 to −0.005 log μg/mL), but was not associated with any of the included EED biomarkers. Contrary to evidence from high-income countries that suggests salivary SIgA increases rapidly with age in young children, the high prevalence of enteric infections may have led to a suppression of immunological development in this study sample and could in part explain the similar SIgA levels between children of different ages.

## 1. Background

In low- and middle-income countries, children experience a high prevalence of enteric infections [1,2] and subsequently suffer disproportionally from diarrheal disease morbidity [3]. Enteric infections are also associated with chronic gastrointestinal health outcomes, such as environmental enteric dysfunction (EED) induced from repeated infections [4]. EED is a subclinical disorder associated with intestinal inflammation and a reduced ability to absorb nutrients [5]. Findings from a recent systematic review suggest that intestinal inflammation is linked to child stunting [6], the burden of which is greatest in low- and middle-income countries [7].

Characterizing enteric infections often relies on the collection of stool samples to use culture-based or molecular methods to detect enteric pathogen shedding [8], or antigen detection assays to measure immunological responses to specific pathogens [9]. Collecting, transporting and storing stool specimens can be resource intensive, so the use of stool samples can be especially challenging for epidemiological studies in resource-constrained settings [10]. Using serum or saliva as biological matrices in immunological assays may present a promising alternative [11]. Serum has the disadvantages that sample collection requires trained individuals, its invasive procedure may lead to low response rates, especially among children [12], and there is a non-zero risk of blood-borne pathogen transmission from respondents to sample collectors [13]. In contrast, saliva collection is non-invasive and requires only minimal training [14], facilitating sample collection in large study populations of young children [15].

One class of biomarkers that can be measured in saliva and may be of interest for gastrointestinal health are salivary antibodies. Different antibody isotypes with varying functions are activated in response to enteric infections. Broadly, immunoglobulin A (IgA) and IgM are produced in response to acute infections, whereas IgG is typically produced later and can be indicative of chronic or historical infections [16]. Saliva has very low concentrations of IgM and IgG antibodies in comparison to IgA [17]. IgA is produced by plasma cells and released in secretory fluids, such as saliva, as secretory IgA (SIgA) [18]. The amount of IgA that humans release is linked to the development of the immune system, which evolves over the course of human life, beginning as an immature immune system during infancy that matures during childhood [19]. Accordingly, previous research on changes in salivary SIgA concentrations with age, suggest a rapid increase during infancy [20], followed by more gradual increases during early childhood, and a stabilization at adult levels during adolescence [21].

The biological mechanisms underlying SIgA-mediated immunity are convoluted, with three separate immunological mechanisms active in protecting the intestinal epithelium from enteric infections. The first prevents pathogens from attaching to the intestinal epithelial cell barrier, most commonly by a process known as immune exclusion [22]. Humans release three grams of SIgA in to the intestinal lumen every day [23], where it acts as the first line of defense to protect the intestinal epithelium against pathogenic organisms [24]. SIgA is transported across the intestinal epithelium and into the lumen by binding to the polymeric immunoglobulin receptor (pIgR), where it binds to the pathogen and neutralizes its ability to attach to the intestinal epithelium and cause infection [25]. The second is specifically targeted at viruses, by neutralizing viruses inside the epithelial cell and preventing assembly/disassembly and exit from the epithelial cell [25]. Third, if damage to the epithelial cell barrier has allowed pathogen invasion in to the lamina propria, SIgA can bind to the pathogen and transport it back across the epithelial boundary using the pIgR for excretion [26].

This study sought to estimate acute antibody responses measured in saliva among children enrolled in the Maputo Sanitation trial (MapSan) in Maputo, Mozambique. Specifically, we quantified associations between salivary SIgA and the number of concurrent enteric infections experienced by young children, as well as concentrations of biomarkers of local gut inflammation and permeability.

## 2. Materials and Methods

### 2.1. Study Setting and Participants

MapSan was a controlled, before-and after trial of an urban sanitation intervention to reduce enteric infections and improve other health metrics in children in Maputo, Mozambique [27]. The study was located in low-income, unplanned settlements and enrolled children aged one to 48 months during the baseline phase between February 2015 and February 2016 and subsequently conducted 12- and 24-month follow-up surveys. Enrollment in MapSan was progressive, and all eligible, consenting children were enrolled during each survey phase. This included children aged 1–48 months at the time of the visit and children under the age of seven years who would have been aged 1–48 months if present and enrolled at baseline. MapSan collected data from 987 children at baseline, 939 at 12-month follow-up, and 996 at 24-month follow-up. For this sub-study, we analyzed one saliva sample per child collected from a subset of 244 children at either 12- or 24-month follow-up.

Saliva samples used for this study were selected based on sufficient sample volume (>10 μL), child age, availability of a matched stool sample, and the number of infections detected in that stool sample. We excluded samples from children under the age of 12 months due to the presence of maternal antibodies and lack of crevicular fluid (saliva excreted between the teeth and gums enriched with Ig) [28]. Breastfeeding is uncommon in children above the age of 12 months in Mozambique [29]. Saliva samples were eligible if they were collected within 10 days of matched stool samples. Due to the high prevalence of enteric infection in the MapSan cohort [30], we selected all eligible saliva samples from children where no infections were detected in matched stool samples.

### 2.2. Procedures

Saliva samples were collected during three cross-sectional household survey visits at the baseline, 12- and 24-month assessments of the MapSan study. Baseline samples were collected with cotton swabs and were subsequently not eligible for this study due to insufficient sample volume. During the 12- and 24-month assessments saliva samples were collected by rubbing Oracol saliva swabs (Malvern Medical Developments, Worcester, United Kingdom), along the child’s gum for one minute to collect crevicular fluid. Samples were transported in a cooler and frozen at −80 °C until processed. To prepare samples for processing, we centrifuged saliva swabs at 2000 rcf for 10 min, before removing saliva from the sample collection tubes and recording sample volume. We excluded saliva samples visibly contaminated with serum (i.e., due to gum bleeding [31]).

We used enzyme-linked immunosorbent assays (ELISA) to process samples for SIgA, in accordance with the manufacturer’s directions (Salimetrics, Carlsbad, CA, USA), and processed 70% of samples in replicate. For replicate samples we excluded results where individual replicates were not within 20% of the replicate mean. Laboratory methods to detect enteric pathogens and EED biomarkers in matched stool samples are reported elsewhere [30]. Briefly, stool samples collected for the MapSan study were analyzed for detection of 14 enteric pathogens using the molecular-based Gastrointestinal Pathogen Panels (GPP, Luminex Corp, Austin, TX, USA). The GPP included bacterial pathogens (*Campylobacter (C. jejuni, C. coli, and C. lari)*, *Clostridium difficile (C. difficile)*, *Escherichia coli (E. coli)* O157, Enterotoxigenic *E. coli* (ETEC), Shiga-like toxin producing *E. coli* (STEC), *Shigella, Vibrio cholerae* *(V. cholerae)* and *Yersinia enterocolitica (Y. enterocolitica)*), protozoan pathogens (*Giardia*, *Cryptosporidium* and *Entamoeba histolytica (E. histolytica)*) and viral pathogens (Adenovirus 40/41, Norovirus GI/GII, and Rotavirus A). The same stool samples were analyzed for biomarkers of gut inflammation and permeability using ELISA assays. Biomarkers included myeloperoxidase and fecal calprotectin, both markers of neutrophil activity [32], alpha-1 antitrypsin, a protein released during inflammation and marker of gut permeability [33], and neopterin, a marker of T helper cell-derived immune activation [34].

### 2.3. Statistical Analysis

We matched SIgA measured in each saliva sample to individual data on enteric pathogens and EED biomarkers detected in stool samples collected within 10 days of saliva samples. Most stool and saliva samples were collected on the same day or within 24 h of each other, and all were modeled as cross-sectional matched samples. To account for non-normality of the data, we log-transformed all SIgA and EED biomarker data. For our primary analyses, we used multilevel linear models to account for potential confounders and model cross-sectional associations between (1) salivary SIgA concentrations and the number of concurrent enteric infections experienced by a child, and (2) salivary SIgA and concentrations of EED biomarkers:

(1) $${log}_{10}\left(SIgA\right)=\beta_{1}\inf\left(nr\right)+\beta_{2}age+\beta_{3}vol+\beta_{4}rain$$

(2) $${log}_{10}\left(SIgA\right)=\beta_{1}{log}_{10}\left(EED_{b}\right)+\beta_{2}age+\beta_{3}vol+\beta_{4}rain+\beta_{5}\inf\left(nr\right)$$

Evidence in the literature suggests that salivary SIgA levels are affected by age [35], salivary flow rate [36] and seasonality [37], so we controlled for child age (in months), sample volume (in μL) and seasonality in all of our models. We controlled for seasonality by splitting cumulative rainfall during the 30 days before saliva sample collection into terciles, where the first tercile (least rain) represents the reference level in our models. Rainfall data were obtained from the National Oceanic and Atmospheric Administration’s National Centers for Environmental Information (https://www.ncdc.noaa.gov/cdo-web/datatools/findstation). We also controlled for the number of concurrent enteric infections in our EED analyses. EED biomarkers, indexed by *b* in the model, were modeled individually. We conducted sensitivity analyses to estimate the effects outliers had on our findings by excluding observations (for both SIgA and EED biomarkers) that were 1.5 interquartile ranges below the lower quartile or above the upper quartile.

### 2.4. Ethics

Field data collection staff obtained written informed consent from the parent or guardian of each study participant. The study protocol was approved by the Comité Nacional de Bioética para a Saúde (CNBS), Ministério da Saúde (333/CNBS/14), the Ethics Committee of the London School of Hygiene and Tropical Medicine (reference #8345), and the Institutional Review Board of the Georgia Institute of Technology (protocol #H15160). The MapSan study is registered at ClinicalTrials.gov (NCT02362932).

## 3. Results

### 3.1. Summary Characteristics

**Figure 1:**
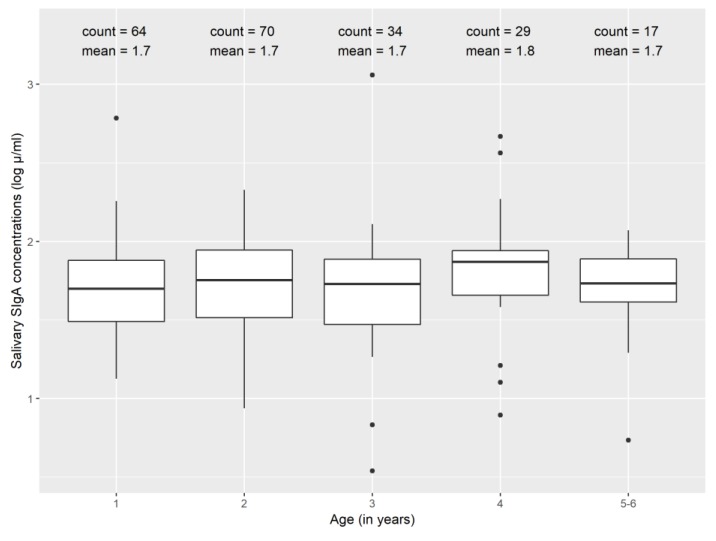
Salivary secretory immunoglobulin A (SIgA) concentrations (log μg/mL) by age.

We extracted 244 saliva samples, 216 samples presenting with sufficient sample volume and no visible blood to be eligible for testing (Table 1). Most of our saliva samples (89%) were collected within one day of stool sample collection. Child age ranged from 1 to 6.7 years with a median age of 2.5 years. Most samples were from children aged 1–2 years (63%) and fewer from children aged 3–6 years (37%). Two samples were excluded from our analyses due to replicate rejection, but otherwise we found acceptable coefficients of variation between replicate samples. We found median salivary SIgA levels of 54 μg/mL (inter-quartile range (IQR): 34, 85 μg/mL) in this study population, and salivary SIgA was similar between children of different ages (Figure 1).

### 3.2. Secretory Immunoglobulin A (SIgA) and Enteric Infections

**Figure 2:**
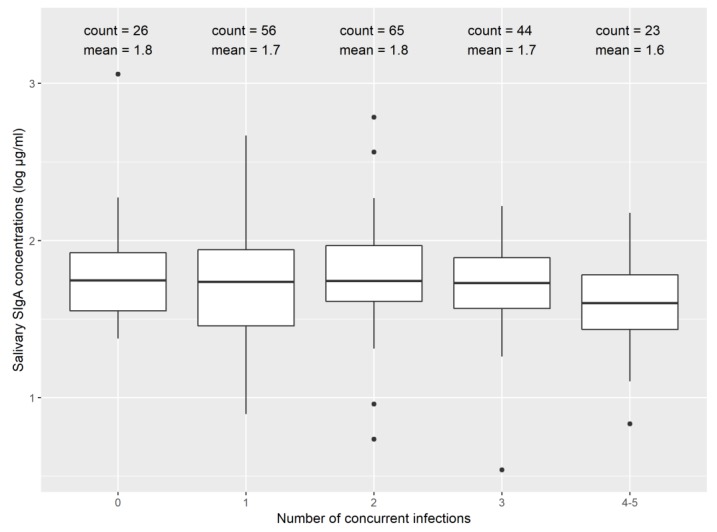
Salivary SIgA concentrations stratified by children experiencing different numbers of concurrent infections.

**Figure A1:**
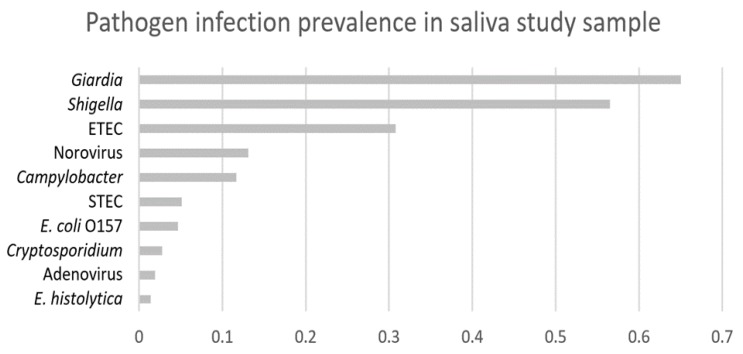
Prevalence of specific pathogens detected in stool samples in the MapSan saliva study sub-sample.

Salivary SIgA concentrations were similar between children experiencing none, one, two, three, or four to five concurrent infections detected in matched stool samples (Figure 2). This was a non-random sample, so the distribution of infections with specific pathogens for this sub-sample was not representative of the distribution found in the MapSan cohort (Figure A1).

Results from our statistical analysis suggested lower salivary SIgA −0.04 log μg/mL (95% confidence interval (CI): −0.08 to −0.005 log μg/mL) for a one unit higher number of concurrent infections experienced by a child, although this association was weaker after removing outliers (Table 2). Sample volume was also significantly negatively associated with salivary SIgA, whereas we found no statistical difference of salivary SIgA with child age or higher cumulative rainfall in the same model.

### 3.3. SIgA and Environmental Enteric Dysfunction (EED) Biomarkers

We found no association between salivary SIgA and EED biomarkers of inflammation and permeability, in models including all samples and after removing outliers (Table 3).

## 4. Discussion

This study measured salivary SIgA and tested its association with enteric infections and EED biomarkers found in matched stool samples from children aged one to six years in low-income urban neighborhoods in Maputo, Mozambique. We found no differences in salivary SIgA concentration between children of different ages and lower SIgA with higher numbers of concurrent infections detected in matched stool samples. We found no relationship between SIgA and EED biomarkers, suggesting that mucosal immune responses in this population were not associated with local gut inflammation or permeability.

Our findings suggest that immunological development might be stifled in a study population of children experiencing a high prevalence of enteric infections and concurrent infections [38]. One possible hypothesis that could explain our findings is that the number of concurrent infections with enteric pathogens may contribute to child malnutrition [39,40] and severe malnutrition is associated with lower levels of SIgA in children [41]. It is notable that in addition to finding a negative relationship between SIgA and the number of concurrent infections, we also found no difference in SIgA concentrations between children of different ages. Age (in months) was not a significant confounder in any of our models, and we also did not see a trend of higher median salivary SIgA concentrations after stratifying by age group (in years). This is in contrast to previous findings from other studies set in high-income countries. Evidence from Estonia, Sweden, Iceland and Israel suggests that salivary SIgA levels are three to four times higher in children aged five to six years compared to one-year old children [21,42,43]. Taken together, these findings suggest that immunological development in this study sample of children living in an urban slum in Maputo, Mozambique, experiencing high rates of enteric infections may be inhibited during early life stages.

The findings from our study need to be interpreted with its limitations in mind. We used a cross-sectional study design so were not able to make comparisons of salivary SIgA between the same population of children at different time points. We found high variability in our SIgA estimates and the negative association between SIgA and the number of concurrent infections was no longer statistically significant after removing outliers. We were limited by sample size for children with no detected enteric infections in matched stool samples, given the high prevalence of any enteric infection (infection with ≥1 enteric pathogen) in the parent study, especially among children older than one year [44]. Approximately two-thirds of our study population were children aged 1–2 years, with a smaller sample of children aged 3–6 years. The challenge associated with high variability of SIgA concentrations in whole saliva between and within individuals has previously been documented [37], and we were not able to validate our salivary findings by measuring serum IgA in parallel. There are a number of external factors that could affect salivary SIgA concentrations that we were not able to control for in our model. These factors include psychological stress [45], diurnal variations [46], child behavior like restlessness and crying [47] and dental health [48]. It is also important to note that we defined infections with enteric pathogens as those detected by the GPP in the MapSan analysis of matched stool samples. Children in our study sample may have been experiencing infections with other pathogens not included in the GPP, recent infections where pathogens had not yet begun shedding in stool and thus were not detectable in the matched stool samples, past infections with continued shedding but no active symptoms, or passage of pathogens detected in the GPPs that did not result in active infection.

## 5. Conclusions

We found that children in a low-income urban slum experiencing a high prevalence of enteric infections did not show differences in salivary SIgA with age found in high-income countries, and may be experiencing a suppression of immunological development during early life stages. Particularly notable were the lower salivary SIgA concentrations with higher numbers of concurrent infections combined with similar concentrations of salivary SIgA between infants and pre-school aged children, a life stage where the immune system is developing and where SIgA levels are expected to increase. Due to the limitations of salivary SIgA diagnostics, including high variability of SIgA levels and a number of external factors we were not able to control for in our analyses, our findings need to be interpreted with caution. Future directions for this research could include measuring IgA in serum in parallel to compare it to salivary findings, and sampling the same children at multiple time points to analyze changes in IgA over time, in a setting where infections with enteric pathogens are common. In this study we were also limited by testing for non pathogen-specific SIgA, and a next step for this research could include the exploration of pathogen-specific salivary SIgA as a biomarker to estimate specific infections [49]. Comparing pathogen-specific antibody responses in saliva to detection of those pathogens in matched stool samples is a research need that has also been outlined by researchers at the US Environmental Protection Agency (EPA) [50].

## Figures and Tables

**Table 1:** Summary characteristics.

Characteristic	
Number of saliva samples	
Extracted	244
Excluded due to insufficient volume	13
Excluded due to visible serum	15
Excluded due to replicate rejection	2
Included in analysis	214
Male child (%)	50
Child age in years—Median (inter-quartile range (IQR))	2.5 (1.8, 3.7)
Difference in days between saliva and stool sample collection—Median (IQR)	0 (−1, 1)
Sample volume available in μL—Median (IQR)	175 (100, 300)
Salivary SIgA levels in μg/mL—Median (IQR)	54 (34, 85)
Coefficient of variation between duplicate samples (%)	6.4

**Table 2:** Difference in salivary SIgA with a higher number of concurrent infections, after controlling for age (in months), sample volume (in μL) and 30-day rainfall (in terciles).

	All Samples (N = 214)			After Removing Outliers (N = 206)		
	Difference in SIgA (log μg/mL)	95% Confidence Interval	*p*-Value	Difference in SIgA (log μg/mL)	95% Confidence Interval	*p*-Value
Number of infections	−0.04	(−0.08, −5 × 10^−3^)	0.03	−0.03	(−0.06, 2 × 10^−3^)	0.07
Age (in months)	4 × 10^−4^	(−2 × 10^−3^, 3 × 10^−3^)	0.79	1 × 10^−3^	(−1 × 10^−3^, 3 × 10^−3^)	0.31
Sample volume (in μL)	−1 × 10^−3^	(−9 × 10^−4^, −3 × 10^−4^)	<0.001	−6 × 10^−4^	(−8 × 10^−4^, −3 × 10^−4^)	<0.001
Rainfall (terciles)	0.03	(−0.02, 0.08)	0.29	0.04	(−4 × 10^−3^, 0.08)	0.07

**Table 3:** Difference in salivary SIgA for a unit difference in environmental enteric dysfunction (EED) biomarker concentration found in stool, after controlling for age, sample volume, 30-day rainfall, and number of concurrent infections.

	All Samples				After Removing Outliers			
EED Biomarker	N	Difference in SIgA (log μg/mL)	95% Confidence Interval	*p*-Value	N	Difference in SIgA (log μg/mL)	95% Confidence Interval	*p*-Value
Neopterin (log nmol/L)	188	0.02	(−0.09, 0.13)	0.75	180	−0.02	(−0.12, 0.07)	0.61
Myeloperoxidase (log ng/mL)	213	0.02	(−0.07, 0.12)	0.64	201	0.04	(−0.05, 0.12)	0.39
Calprotectin (log ng/mL)	211	0.02	(−0.06, 0.10)	0.68	202	4 × 10^−3^	(−0.07, 0.07)	0.91
Alpha-1 antitrypsin (log ng/mL)	207	−0.08	(−0.17, 4 × 10^−3^)	0.06	196	−0.02	(−0.1, 0.06)	0.62
